# Submucosal abscess at the esophagogastric junction successfully treated by endoscopic submucosal dissection: A rare case report

**DOI:** 10.1055/a-2641-2012

**Published:** 2025-07-25

**Authors:** Lihua Guo, Jiaxin Ge, Jinfeng Wen, Lijiang Huang, Fang Huang, Mengpei Zhang, Guo-Liang Ye

**Affiliations:** 1 117881 Department of Gastroenterology, The First Affiliated Hospital of Ningbo University, Ningbo, China; 2 Department of Gastroenterology, Xiangshan First People’s Hospital Medical and Health Group, Ningbo, China

A 53-year-old woman was admitted to the hospital two days ago presenting with fever, upper abdominal pain, and dysphagia. Six months ago, she was admitted to the local hospital, where a gastroscopy revealed a submucosal abscess of the esophagogastric junction ( Video 1 ). Following endoscopic needle aspiration for pus drainage, her condition improved ( Fig. 1 ). Two days ago, her aforementioned symptoms recurred and worsened, accompanied by a fever with a peak temperature of 38.1 °C. The laboratory tests indicated an elevated inflammatory response.

Endoscopic submucosal dissection successfully treated a submucosal abscess at the esophagogastric junction.Video 1

**Fig. 1:**
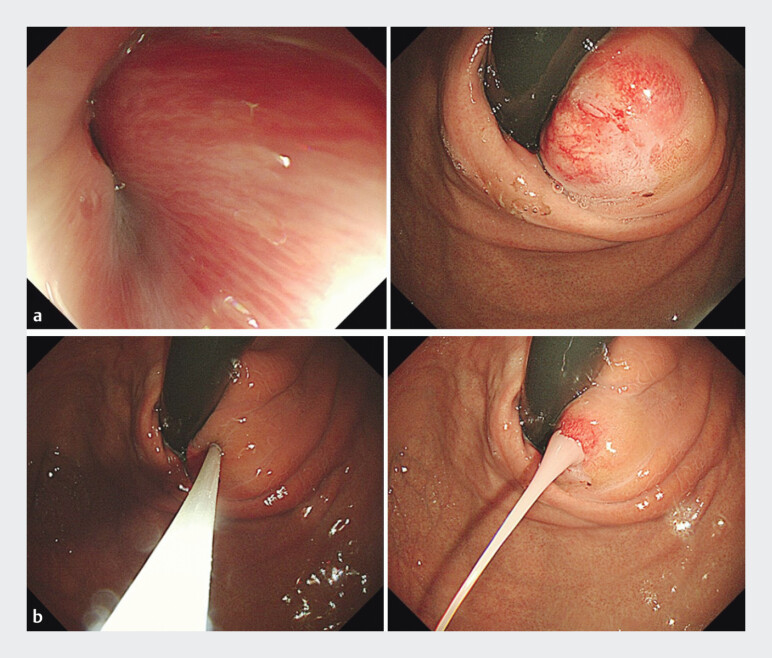
**a** Endoscopic examination revealed a submucosal abscess at the esophagogastric junction. **b** Fine-needle aspiration was conducted to promote effective drainage.

A contrast-enhanced computed tomography (CT) scan of the abdomen revealed thickening of the lower esophagogastric wall along with a low-density focus, which may indicate the potential formation of an abscess ( Fig. 2 ). Antibiotic therapy was initiated with 2 g cefoperazone-sulbactam every 8 hours (intravenous drip). White-light endoscopy revealed a mucosal bulge in the esophagogastric junction. Endoscopic ultrasonography (EUS) revealed a submucosal hypoechoic mass measuring approximately 13 mm in diameter, with the muscular layer integrity remaining intact ( Fig. 3 ). The patient then underwent endoscopic submucosal dissection (ESD) of the suspected abscess. The excised lesion specimens from the wound were fixed with pins and subsequently sent for pathological examination. The patient was discharged with no further symptoms after 5 days of treatment. Pathology revealed a suppurative inflammation and reactive changes in focal squamous epithelium ( Fig. 4 ). On follow-up endoscopy three months later, there was healing mucosa without submucosal lesions.

**Fig. 2:**
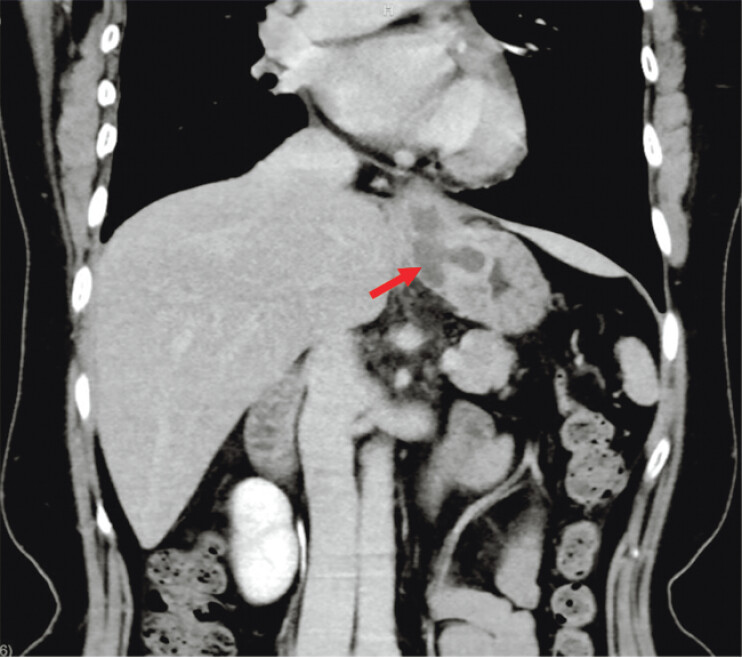
Contrast-enhanced computed tomography of the abdomen with coronal reconstruction demonstrated wall thickening at the esophagogastric junction and the potential formation of an abscess (red arrow).

**Fig. 3:**
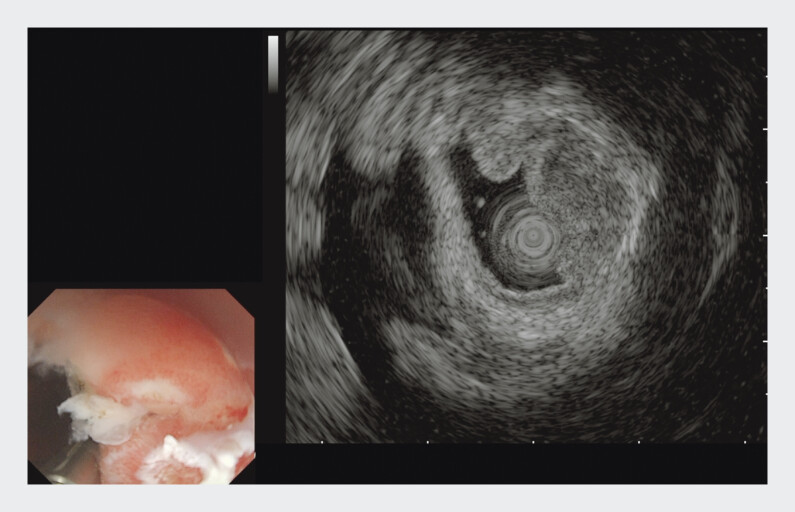
Endoscopic ultrasound revealed a suspected abscess at the esophagogastric junction.

**Fig. 4:**
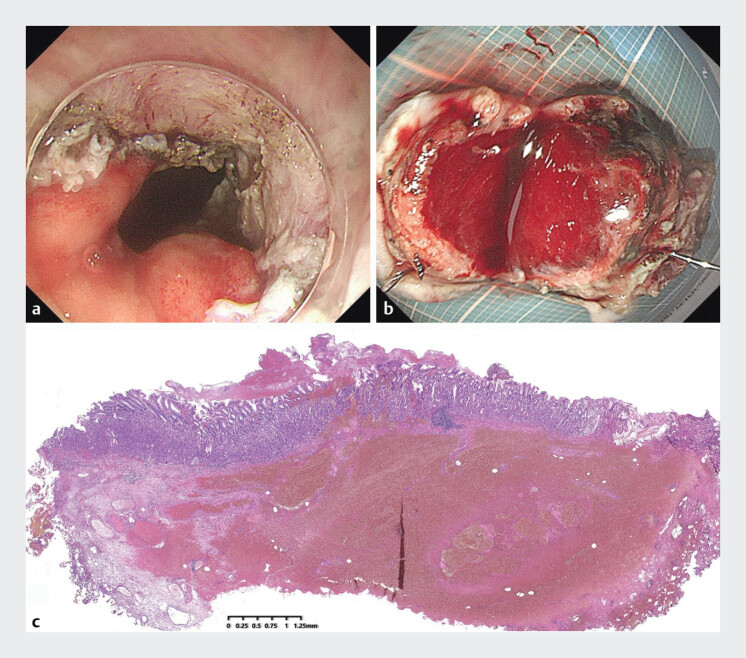
**a** The lesion was completely removed. **b** A hematoma was observed following incision of the lesion. **c** The pathological findings indicated the presence of purulent inflammation with hemorrhage.

The formation of a submucosal abscess in the esophagus or stomach is typically associated with foreign bodies or a condition of iatrogenic etiology 1 2 . Prompt diagnosis and early therapy are needed to prevent death and prolonged serious illness; the key to diagnosis is an awareness of frequent atypical presentations 3 . The results showed that endoscopic puncture and drainage of esophagogastric junction abscesses could only temporarily relieve the symptoms, while ESD could cure submucosal abscesses of the esophagogastric junction.

Endoscopy_UCTN_Code_TTT_1AO_2AN

Endoscopy_UCTN_Code_TTT_1AO_2AG_3AD

Endoscopy_UCTN_Code_CCL_1AB_2AD_3AC

## Notes

Endoscopy E-Videos https://eref.thieme.de/e-videos
*E-Videos* is an open access online section of the journal *Endoscopy* , reporting on interesting cases and new techniques in gastroenterological endoscopy. All papers include a high-quality video and are published with a Creative Commons CC-BY license. Endoscopy E-Videos qualify for HINARI discounts and waivers and eligibility is automatically checked during the submission process. We grant 100% waivers to articles whose corresponding authors are based in Group A countries and 50% waivers to those who are based in Group B countries as classified by Research4Life (see: https://www.research4life.org/access/eligibility/ ). This section has its own submission website at https://mc.manuscriptcentral.com/e-videos .
